# Conditional Mutation of Hand1 in the Mouse Placenta Disrupts Placental Vascular Development Resulting in Fetal Loss in Both Early and Late Pregnancy

**DOI:** 10.3390/ijms22179532

**Published:** 2021-09-02

**Authors:** Jennifer A. Courtney, Rebecca L. Wilson, James Cnota, Helen N. Jones

**Affiliations:** 1 Center for Fetal and Placental Therapy, Cincinnati Children’s Hospital Medical Center, Cincinnati, OH 45229, USA; jcourtneymdb@gmail.com; 2 Center for Research in Perinatal Outcomes, College of Medicine, University of Florida, Gainesville, FL 32603, USA; rebecca.wilson@ufl.edu; 3 Department of Physiology and Functional Genomics, College of Medicine, University of Florida, Gainesville, FL 32603, USA; 4 Heart Institute, Cincinnati Children’s Hospital Medical Center, Cincinnati, OH 45229, USA; James.Cnota@cchmc.org

**Keywords:** congenital heart disease, placenta, hand1, vascular development, pregnancy

## Abstract

Congenital heart defects (CHD) affect approximately 1% of all live births, and often require complex surgeries at birth. We have previously demonstrated abnormal placental vascularization in human placentas from fetuses diagnosed with CHD. *Hand1* has roles in both heart and placental development and is implicated in CHD development. We utilized two conditionally activated *Hand1^A126fs/+^* murine mutant models to investigate the importance of cell-specific *Hand1* on placental development in early (*Nkx2-5^Cre^*) and late (*Cdh5^Cre^*) pregnancy. Embryonic lethality occurred in *Nkx2-5^Cre^/Hand1^A126fs/+^* embryos with marked fetal demise occurring after E10.5 due to a failure in placental labyrinth formation and therefore the inability to switch to hemotrophic nutrition or maintain sufficient oxygen transfer to the fetus. Labyrinthine vessels failed to develop appropriately and vessel density was significantly lower by day E12.5. In late pregnancy, the occurrence of *Cdh5^Cre+^;Hand1^A126fs/+^* fetuses was reduced from 29% at E12.5 to 20% at E18.5 and remaining fetuses exhibited reduced fetal and placental weights, labyrinth vessel density and placenta angiogenic factor mRNA expression. Our results demonstrate for the first time the necessity of *Hand1* in both establishment and remodeling of the exchange area beyond early pregnancy and in patterning vascularization of the placental labyrinth crucial for maintaining pregnancy and successful fetal growth.

## 1. Introduction

Congenital heart disease (CHD) is the most common birth defect, affecting ~1% of all live births [1]. Babies born with a CHD often undergo corrective surgeries within days of being born, and survival from these surgeries often depends on size at birth [2]. Population studies have demonstrated that pregnancies complicated by CHD carry a higher risk of developing pathologies associated with abnormal placental development and function including growth disturbances [3,4,5], preeclampsia [6,7,8,9], preterm birth [10,11], and stillbirth [12]. The placenta serves as the mediator between mother and developing fetus to provide nutrition and gas exchange, remove fetal wastes, and prevents mixing of maternal and fetal blood [13]. It is unsurprising, while relatively recently demonstrated, that placental development is often affected in pregnancies with CHD as both the heart and placenta are vascular organs that develop concurrently very early in gestation, and shared pathways direct the development of both [14]. Placentas of fetuses with CHD often exhibit changes that disrupt the proper patterning and function of the maternal/fetal exchange area including underdeveloped vasculature and impaired nutrient transport [15,16].

Genome-wide associations studies (GWAS) undertaken previously have identified almost 400 genes associated with CHD [17,18,19]. However, despite decades of research with a primary focus on genetic etiology, the underlying cause of these CHD remains unknown in the majority of cases [20]. Additionally, modeling studies implicating genes and/or environmental influences responsible for abnormal heart development [21] often overlook the involvement of extraembryonic tissues or circumvent it by using ‘cardiac-specific’ conditional knockout mouse models. However, this does not truly reflect the situation in cases of CHD where genetic perturbations would occur in all cells/tissues expressing that gene and many of the identified genes are expressed in other cell types in addition to those found in the heart. Importantly, it has been shown that 68% of 103 knockout models of heart development that exhibited embryonic lethality at or after mid-gestation had abnormal placental development not previously investigated [22]. Such outcomes highlight the strong correlation between placental dysmorphology, angiogenesis and heart development.

One example of a gene involved in both cardiac and placental development is *Hand1* [23]. *Hand1* is a basic helix-loop-helix transcription factor found in multiple organ systems during embryogenesis. *Hand1*-null mice are embryonic lethal by E8.5 due to extraembryonic defects in the yolk sac, chorion, allantois, and trophoblast giant cells [23,24,25,26]. Lineage tracing verified *Hand1* expression at E9.5 in these tissues [27]. Placental labyrinth vascular endothelium also expressed *Hand1* at E14.5 as well as the endothelium of the umbilical vein [27]. Early pregnancy lethality of the global *Hand1* knockout models increases the difficulty in studying its role on placental development as the developing conceptuses are lost before the placenta has fully formed. Furthermore, the relevance of the disruption to placentation by trophoblast giant cell failure is controversial in its applicability to the development of the human placenta due to species differences. There are a number of conditional knockout mouse models in which gene disruption is isolated within the fetus, which, depending on the Cre-driver, show varying developmental outcomes from mid-gestation lethality to viable offspring with mild phenotypes [28,29]. However, these studies focused solely on the fetus, despite the fact that some of the Cre-drivers function in extra-embryonic cells of the yolk sac and placenta, which may explain the wide disparities in outcomes.

The implications for *Hand1* on heart development have been well characterized [23], however currently, there have been no investigations into the placental contribution to embryonic lethality. In the current study, we aimed to determine the placental contribution to embryonic lethality in the *Hand1*-mutant mice in early and late pregnancy using two conditional activation mouse models. Nkx2-5 is a well-known cardiac development gene, but is also required for yolk sac angiogenesis and is expressed in endothelial and hematopoietic cells within yolk sac mesoderm [30,31,32]. Previous investigations using the conditional activation knock-in *Hand1^SFA126fs^* mice generated with *Nkx2-5^Cre^* have shown that *Hand1^A126fs/+^* fetuses die at embryonic day 15.5 (E15.5) and display outflow tract abnormalities, thin myocardium and ventricular septal defects [28]. However, the extraembryonic tissue was not investigated. Cadherin 5 (Cdh5) is an endothelial cell specific gene expressed in the placental fetal vessels. Thus, the use of the *Nkx2-5^Cre^* allows for the study of mutating *Hand1* from E8.5 in the yolk sac and labyrinth trophoblast progenitor cells at a time essential for labyrinth establishment and the switch from histiotrophic to hemotrophic nutrition. The *Cdh5^Cre^* model on the other hand, targets the requirements for *Hand1* in placental vascular branching in pregnancy when *Hand1* is expressed in endothelial cells from E12.5.

## 2. Results

### 2.1. Nkx2-5-Cre Is Expressed in Yolk Sac, Trophoblast Cells, and Cardiomyocytes

**Figure 1:**
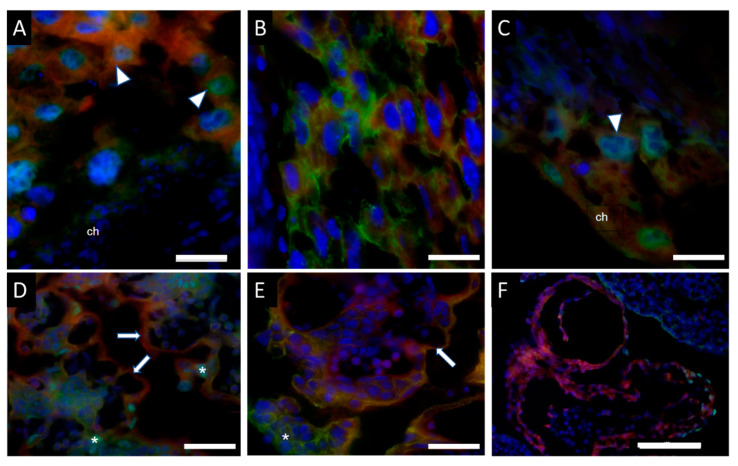
Expression of Nkx2-5-Cre and Hand1 or Cytokeratin 7 in the placenta and fetal heart at embryonic day 8.5 (E8.5), E9.5 and E10.5. (**A**) *Nkx2-5^Cre^* (red) is expressed in trophoblast progenitor cells at E8.5, overlapping with Hand1 protein expression, but not in chorion which does express Hand1; (**B**) Colocalization of *Nkx2-5^Cre^* (red) expression with cytokeratin 7 (green) at E8.5; (**C**) At E9.5, *Nkx2-5^Cre^* (red) and Hand1 (green) are co-expressed in both chorion and labyrinth trophoblast progenitor cells; (**D**) Hand1 (green) and *Nkx2-5^Cre^* (red) overlap in syncytiotrophoblasts of the placental labyrinth, whilst sinusoidal giant cells do not express *Nkx2-5^Cre^* at E10.5; (**E**) Colocalization of *Nkx2-5^Cre^* (red) expression with cytokeratin 7 (green) at E10.5. (**F**) Hand1 is co-expressed in a subset of ventricular cardiomyocytes at E9.5. Arrowhead is trophoblast progenitor cell. Arrow is syncytiotrophoblast. Asterisk is sinusoidal giant cell. ch = chorion. Scale bar = 50 µm (**A**–**E**) and 100 µm (**F**).

Conditionally activated *Hand1^A126FS/+^* females were time mated with homozygous *Nkx2-5^Cre^* males to produce litters containing *Nkx2-5^Cre^;Hand1^A126FS/+^* and *Nkx2-5^Cre^;Hand1^+/+^* embryos. In order to verify the efficacy and spaciotemporal expression of the *Nkx2-5^Cre^*, males were first mated with homozygous tdTomato reporting females. In control mice, immunofluorescence and microscopy confirmed trophoblast expression of the Nkx2-5-Cre at E8.5 as well as Hand1 expression in the trophoblast nucleus (Figure 1A); co-localization of Nkx2-5-Cre expression and the trophoblast marker cytokeratin 7 further confirmed trophoblast expression (Figure 1B). Co-expression of Nkx2-5-Cre and Hand1 in yolk sac, labyrinth trophoblast progenitor cells and syncytiotrophoblast was also confirmed at E9.5 (Figure 1C) and E10.5 (Figure 1D). Sinusoidal giant cells did not express Nkx2-5-Cre at E10.5 but did express cytokeratin 7 (Figure 1E). Analysis of the heart at E9.5 showed strong expression for Nkx2-5-Cre, and Hand1 was localized in the cardiac ventricular tissue (Figure 1F). 

### 2.2. Hand1 Disruption under Nkx2-5-Cre Results in Fetal Demise

**Figure 2:**
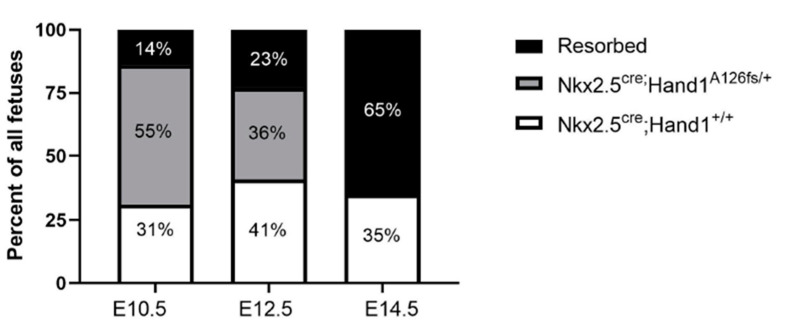
Percentages of resorptions (black), *Nkx2-5^Cre^;Hand1^A126fs/+^* (grey) and *Nkx2-5^Cre^; Hand1^+/+^* (white) embryos in litters recovered at embryonic day 10.5 (E10.5), E12.5 and E14.5. From E10.5, embryos with the *Nkx2-5^Cre^;Hand1^A126fs/+^* genotype show increased fetal demise. *Nkx2-5^Cre^;Hand1^A126fs/+^* fetuses were overrepresented at E10.5 (55%); however, they showed a marked decrease at E12.5 (36%) and no viable fetuses were recovered at E14.5. *n* = 4–8 litters.

*Hand1^A126FS/+^* females mated with *Nkx2-5^Cre^* males were sacrificed at E8.5, E9.5, E10.5, E12.5 and E14.5, and fetuses and placentas collected. The expected ratio of *Nkx2-5^Cre^;Hand1^+/+^* to *Nkx2-5^Cre^;Hand1^A126fs/+^* was 50% each. While *Nkx2-5^Cre^;Hand1^+/+^* littermates showed consistent survival at all timepoints, marked fetal demise occurred after E10.5 in *Nkx2-5^Cre^;Hand1^A126fs/+^* fetuses (Figure 2) and none survived to E14.5. *Nkx2-5^Cre^;Hand1^A126fs/+^* fetuses were overrepresented at E10.5 (55%); however, they showed a marked decrease at E12.5 (36%) and no viable fetuses were recovered at E14.5. Resorptions were not genotyped due to a lack of available fetal tissue.

### 2.3. Early Placental Morphogenesis Is Impaired in the Nkx2-5^cre^;Hand1^A126fs/+^ Implantation Sites

**Figure 3:**
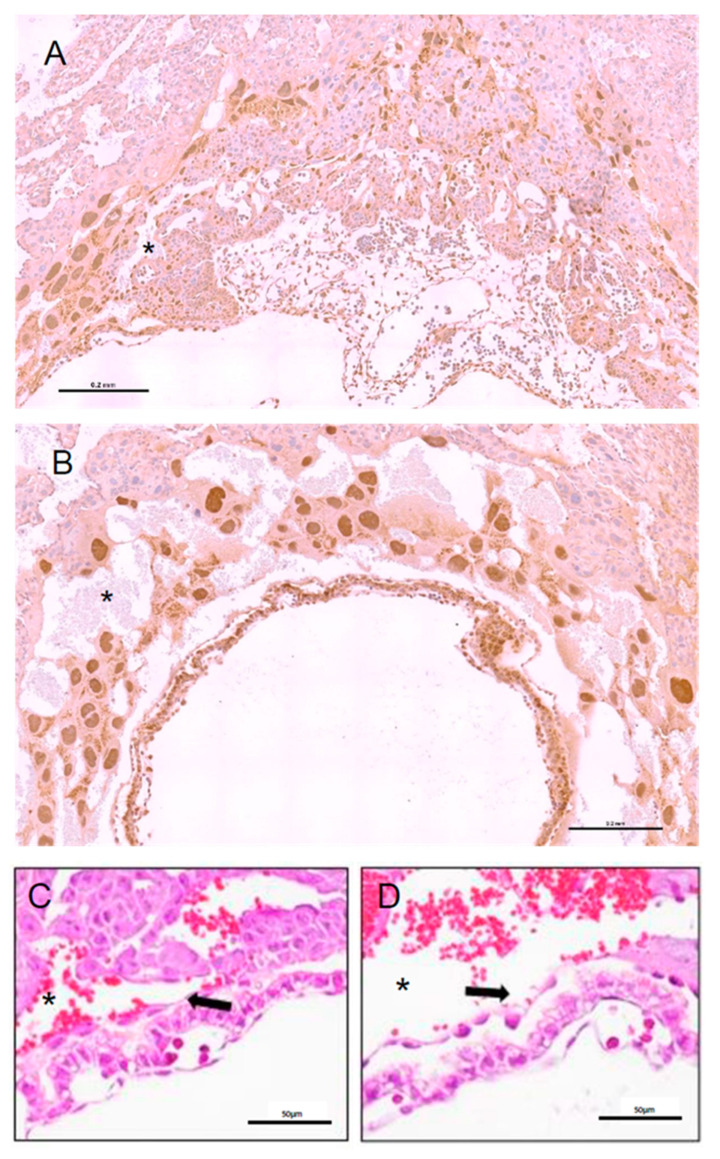
Placenta morphogenesis is impaired as early as embryonic day 9.5 (E9.5) in *Nkx2-5^Cre^;Hand1^A126fs/+^* implantations. (**A**) Control littermate placentas (*Nkx2-5^Cre^;Hand1^+/+^*) exhibit invagination of the chorion and development of an early labyrinth at E9.5; (**B**) In contrast, *Nkx2-5^Cre^;Hand1^A126fs/+^* placentas lack a chorionic plate and only have a disorganized layer of trophoblast giant cells surrounding the amniotic cavity; (**C**) High magnification of the yolk sac morphology in the normal littermates show adjacent chorion and yolk sac; (**D**) The *Nkx2-5^Cre^;Hand1^A126fs/+^* placentas lacked a chorionic layer and yolk sacs were detached from the trophoblast layer separated by large maternal blood spaces. Arrows indicate attachment/detachment of the yolk sac. Asterisk indicate maternal blood spaces. (**A**,**B**) are immunostained for Hand1; scale bar = 0.2 mm; (**C**,**D**) are stained with hematoxylin and eosin; scale bar = 50 µm.

Lack of viable *Nkx2-5^Cre^;Hand1^A126fs/+^* fetuses by E14.5 indicated impaired early placental morphogenesis. Analysis of control litter mate implantation sites at E9.5 revealed invagination and folding of the chorion with scattered Hand1-positive labyrinthine progenitor trophoblasts and a single layer of trophoblast giant cells separating labyrinth from decidua (Figure 3A). Conversely, *Nkx2-5^Cre^;Hand1^A126fs/+^* implantation sites had a disorganized layer of Hand1-positive trophoblast giant cells but lacked Hand1-positive labyrinthine progenitor trophoblasts (Figure 3B). While maternal erythrocytes were present in both genotypes in the developing labyrinthine area, maternal blood spaces were dilated in the conditional activated compared to control littermates and structural integrity of the area disrupted. In addition, yolk sac morphology appeared abnormal in *Nkx2-5^Cre^;Hand1^A126fs/+^* fetuses at E9.5. The yolk sac was adjacent to the chorionic plate in the control littermates (Figure 3C), but was separated from the trophoblast layer in *Nkx2-5^Cre^;Hand1^A126fs/+^* fetuses (Figure 3D).

### 2.4. Nkx2-5^Cre^;Hand1^A126fs/+^ Placentas Exhibit Failed Labyrinthine Vascularization and Syncytiotrophoblast Differentiation

**Figure 4:**
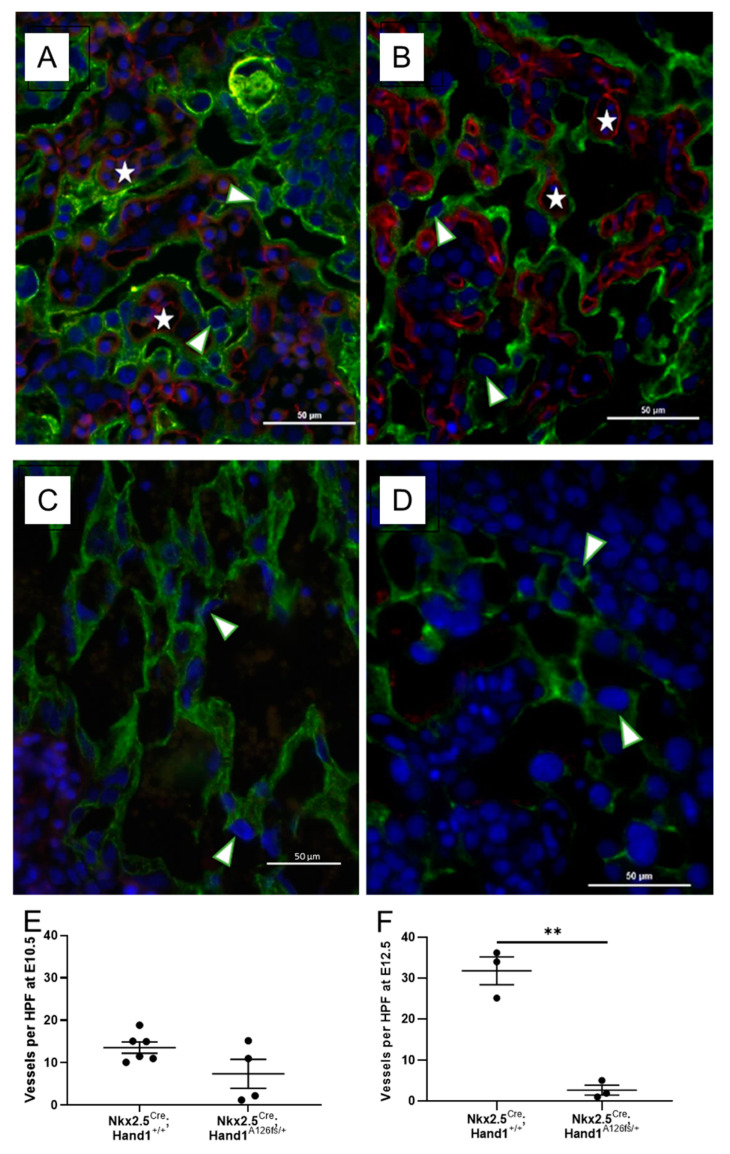
Conditional activation of mutant Hand1 in the placenta results in failed labyrinthine vascular development by embryonic day 12.5 (E12.5). (**A**,**B**) Control (*Nkx2-5^Cre^;Hand1^+/+^*) placentas exhibited normal development of the fetal vasculature with fetal vessels (Red/Star; CD-31 positive) clearly separated from maternal blood space by trophoblast (Green; Cytokeratin-7 positive) at E10.5 (**A**) and E12.5 (**B**); (**C**,**D**) *Nkx2-5^Cre^;Hand1^A126fs/+^* labyrinths however, had very few fetal labyrinthine blood vessels at both E10.5 (**C**) and E12.5 (**D**) and showed disorganization of the trophoblasts with very few remaining cytokeratin-7-positive trophoblasts remaining at E12.5; (**E**) Whilst not statistically significant, fetal vessel counts at E10.5 confirmed a decreased number of fetal vessels (*P* = 0.088); (**F**) There was significantly reduced by E12.5 (*P* = 0.001). Arrowhead indicates sinusoidal trophoblast giant cells. Nuclei are labelled Blue with DAPI. (**A**–**D**) are representative images. (**E**,**F**) individual dots represent average per litter. Data are mean ± SEM. *n* = 3–6 litters. Statistical significance determined using a students *t*-test. ** *P* = 0.001.

Further analysis to identify trophoblast (cytokeratin 7) and fetal vessels (CD-31) in the *Nkx2-5^Cre^;Hand1^A126fs/+^* placentas at E10.5 and E12.5 showed developmental failure of labyrinthine vasculature. CD-31 positive labyrinthine vessels were visible in the control littermate placentas at E12.5 and E14.5 (Figure 4A,B, respectively). Both syncytium and sinusoidal cytokeratin-7-positive trophoblast giant cells were present in the control labyrinth with clear delineation of fetal vasculature from maternal blood spaces (Figure 4A,B). In contrast, very few labyrinthine blood vessels were observed in the *Nkx2-5^Cre^;Hand1^A126fs/+^* placentas at either timepoint (Figure 4C,D). Labyrinthine vessel density trended lower (*P* = 0.088; Figure 4E) in the conditional activated placentas at E10.5, and by E12.5, vessel counts were significantly lower in *Nkx2-5^Cre^;Hand1^A126fs/+^* labyrinths compared to control (*P* = 0.001; Figure 4F). Additionally, the labyrinths at E10.5 showed disorganization of the trophoblasts and lack of syncytial layers while retaining sinusoidal trophoblast giant cells, demonstrating a lack of differentiation along the syncytiotrophoblast lineage (Figure 4C). By E12.5, *Nkx2-5^Cre^;Hand1A^126fs/+^* placentas had very few remaining cytokeratin-7-positive trophoblasts (Figure 4D). There were no significant sex differences identified in labyrinth morphology, fetal vessel density or survival.

### 2.5. Conditional Activation of Mutant Hand1 in Placental Endothelium Resulted in a Reduced Percentage of Cdh5^Cre+^;Hand1^A126fs/+^ Fetuses by E18.5

**Figure 5:**
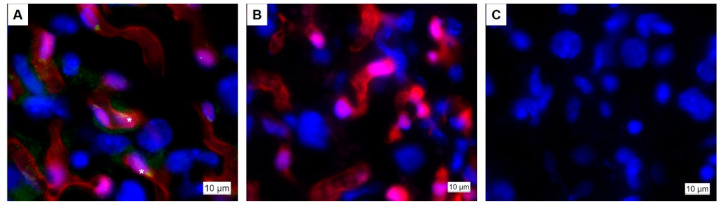
Expression of Cdh5-Cre and Hand1 in the placental labyrinth at embryonic day 16.5. (**A**) Cdh5-Cre (red) is expressed in endothelial cells, overlapping with Hand1 protein expression (green); (**B**) Representative negative control for Hand1 immunofluorescence; (**C**) Representative image of tdTomato fluorescence in a *Cdh5^Cre−^* placenta. Asterix indicates fetal capillary.

**Figure 6:**
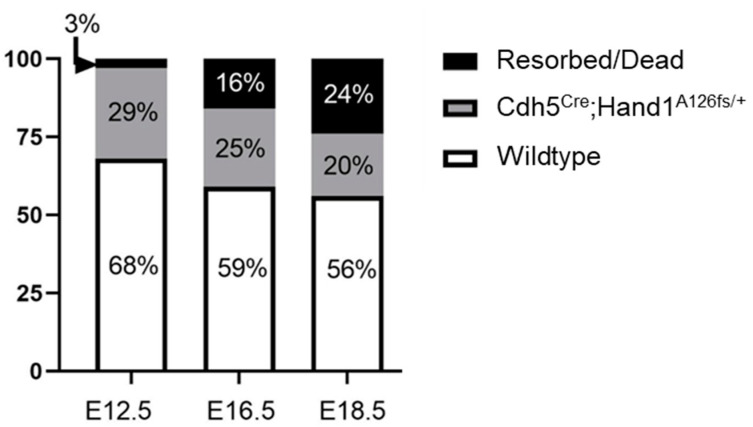
Percentage of resorptions (black), *Cdh5^Cre+^;Hand1^A126fs/+^* (grey) and wildtype (white) fetuses in litters recovered at embryonic day 12.5 (E12.5), E16.5 and E18.5. As gestation progressed, the percentage of resorptions/dead fetuses increased while the percentage of *Cdh5^Cre^;Hand1^A126fs/+^* fetuses decreased. Numbers in parentheses are the total number of pups at each timepoint. *n* = 3–8 dams.

Having confirmed the requirement for *Hand1* to be expressed in yolk sac and labyrinth trophoblast progenitor cells during early pregnancy on successful development of the labyrinth exchange area, we chose to further assess the requirement for *Hand1* in later pregnancy, examining vascular remodeling and expansion in the *Cdh5^Cre+^;Hand1^A126fs/+^*. Mating the *Cdh5^Cre^* mice with tdTomato reporting mice confirmed Cdh5-Cre and Hand1 expression in the endothelial cells of the placental labyrinth at GD16.5 (Figure 5). Conditional activated *Hand1^A126fs/+^* females were then time mated to hemizygous *Cdh5^Cre^* males to produce *Cdh5^Cre+^;Hand1^A126fs/+^*, *Cdh5^Cre+^;Hand1^+/+^*, *Cdh5^Cre−^;Hand1^A126fs/+^* and *Cdh5^Cre−^;Hand1^+/+^* fetuses. Fetuses and placentas were harvested at E12.5, E16.5 and E18.5. At E12.5, there was a 3% resorption rate which increased to 16% at E16.5 and to 24% at E18.5 (Figure 6). Meanwhile, the percentage of *Cdh5^Cre+^;Hand1^A126fs/+^* fetuses was reduced from 29% at E12.5 to 25% at E16.5 and 20% at E18.5.

### 2.6. Fetal and Placental Weights Were Reduced in Cdh5^cre^; Hand1^A126fs/+^ by E18.5

**Figure 7:**
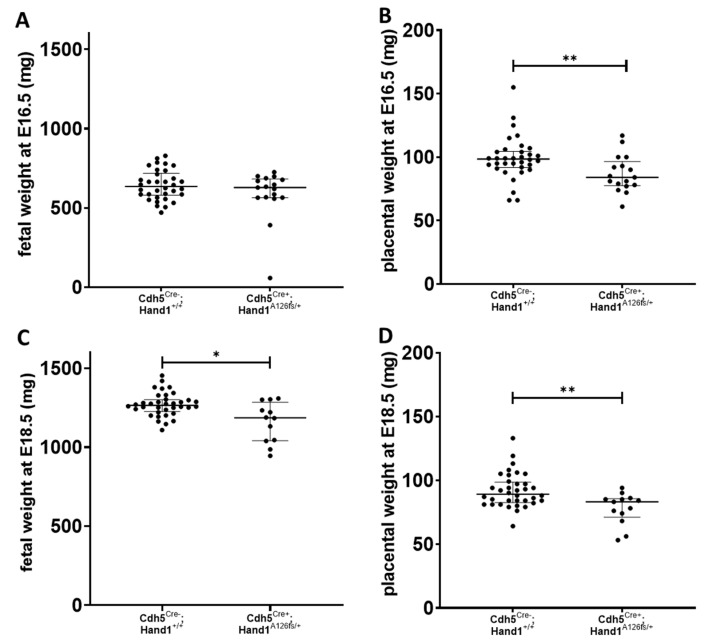
Effect of conditionally activating mutant Hand1 in placental fetal endothelium in late pregnancy. (**A**) At embryonic day 16.5 (E16.5) fetal weight was not different between *Cdh5^Cre+^;Hand1^A126fs/+^* and control (*Cdh5^Cre−^;Hand1^+/+^*) littermates; (**B**) However, placental weight at E16.5 was significantly lower in the *Cdh5^Cre+^;Hand1^A126fs/+^* fetuses; (**C**,**D**) By E18.5, both fetal (**C**) and placental (**D**) weight was significantly lower in the *Cdh5^Cre+^;Hand1^A126fs/+^* fetuses when compared to the control littermates. Data are median ± interquartile range. Individual dots represent individual fetuses or placentas. *n* = 8 litters per time point. Statistical significance determined using a Mann–Whitney test. * *P* < 0.05; ** *P* < 0.01.

At E16.5, no difference in fetal weight was found between *Cdh5^Cre+^;Hand1^A126fs/+^* and *Cdh5^Cre−^;Hand1^+/+^* littermates (*P* > 0.05; Figure 7A), however, placental weight of the *Cdh5^Cre+^;Hand1^A126fs/+^* fetuses was significantly lower (*P* = 0.01; Figure 7B). By E18.5, both fetal and placental weight were lower in the *Cdh5^Cre+^;Hand1^A126fs/+^* genotype when compared to their *Cdh5^Cre−^;Hand1^+/+^* littermates (*P* < 0.001 and *P* = 0.002; Figure 7C,D, respectively). There was no difference in fetal or placental weight between males and females in either genotype.

### 2.7. Labyrinthine Vessel Density and Angiogenic Factor mRNA Expression Was Significantly Reduced in the Cdh5^Cre+/−^;Hand1^A126fs/+^ Placentas

**Figure 8:**
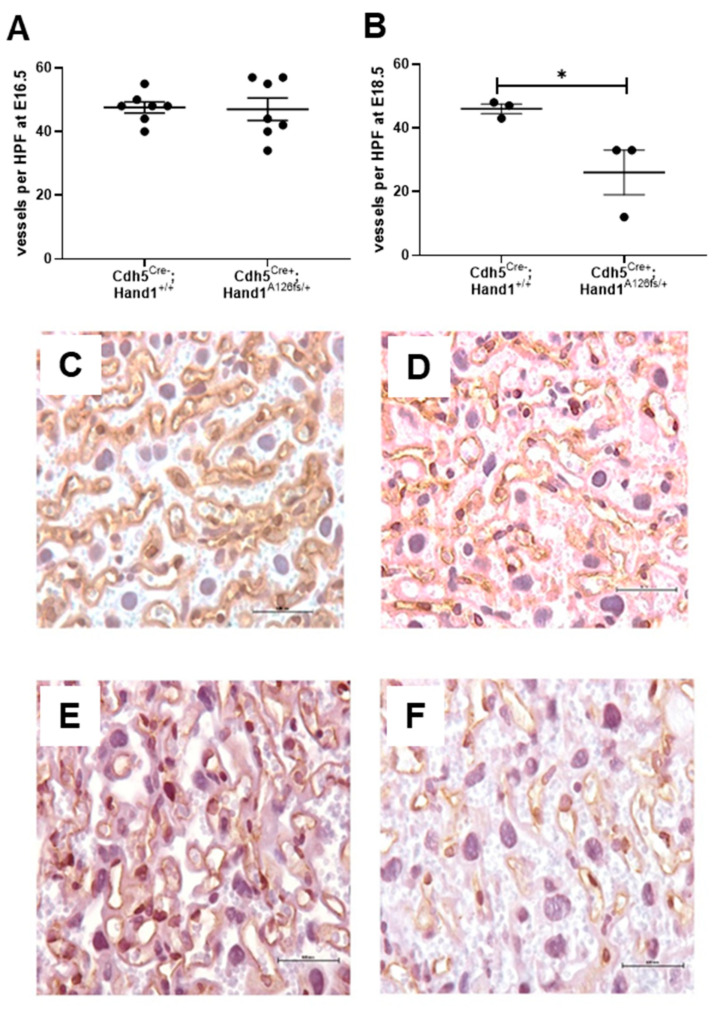
Fetal vessel density in the placenta labyrinth at embryonic day 16.5 (E16.5) and E18.5 between wildtype (*Cdh5^Cre−^;Hand1^+/+^*) and *Cdh5^Cre+^;Hand1^A126fs/+^* placentas. (**A**) At E16.5 there was no difference in the number of fetal vessels per high powered field (HPF) between *Cdh5^Cre−^;Hand1^+/+^* and *Cdh5^Cre+^;Hand1^A126fs/+^* placentas; (**B**) At E18.5, the number of fetal vessels per HPF was reduced in the labyrinth of the *Cdh5^Cre+^;Hand1^A126fs/+^* placentas compared to wildtype; (**C**,**D**) are representative images at E16.5 and E18.5 of a *Cdh5^Cre−^;Hand1^+/+^* placenta, respectively. (**E**,**F**) are representative images at E16.5 and E18.5 of a *Cdh5^Cre+^;Hand1^A126fs/+^* placenta, respectively. Data are mean ± SEM; (**A**,**B**) individual dots represent average per litter. *n* = 3–4 litters per time point. Statistical significance determined using a students *t*-test. * *P* < 0.05. CD-31 staining (brown) with hematoxylin counterstain. Scale bars = 500 µm.

**Figure 9:**
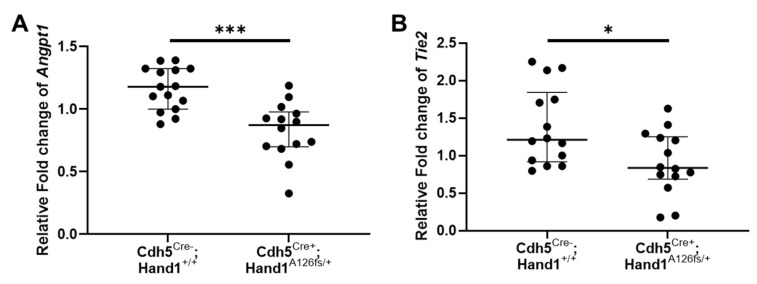
Analysis of angiogenic factor *Angiopoietin 1* (*Angpt1*) and *Angpt1 Receptor* (*Tie2*) mRNA expression in wildtype (*Cdh5^Cre−^;Hand1^+/+^*) and *Cdh5^Cre+^;Hand1^A126fs/+^* placentas at embryonic day 16.5. mRNA expression of both Angpt1 (**A**) and Tie2 (**B**) was reduced in the *Cdh5^Cre+^;Hand1^A126fs/+^* placentas compared to wildtype. Expression was not different between the fetal sexes for either genotype. Data are median ± interquartile range. Individual dots represent individual placentas. *n* = 3–6 litters per time point. Statistical significance determined using a Mann–Whitney test. * *P* < 0.05, *** *P* < 0.001.

Using immunohistochemistry, CD-31 positive fetal vessels in the placental labyrinth were identified and counted to assess placental vascularization at E16.5 and E18.5. There was no difference in in the number of labyrinth vessels in the placenta between *Cdh5^Cre+^;Hand1^A126fs/+^* and littermate controls at E16.5 (Figure 8A). However, by E18.5, there was a significant reduction in the number of vessels in the labyrinth of the *Cdh5^Cre+^;Hand1^A126fs/+^* fetuses (*P* < 0.05; Figure 8B). Whilst vascular density was not reduced in the *Cdh5^Cre+^;Hand1^A126fs/+^* placentas at E16.5, there was a reduction in the mRNA expression of *Angiopoietin 1* (*Angpt1*) and *Angpt1 Receptor* (*Tie2*) in the placentas of the *Cdh5^Cre+^;Hand1^A126fs/+^* at E16.5 when compared to littermate controls (*P* < 0.001 and *P* = 0.012; Figure 9A,B, respectively). mRNA expression of *Angpt2*, *Vascular endothelial growth factor α* (*Vegfα*) and *Placenta growth factor* (*Plgf*) were also assessed however, there was no difference between the genotypes (Data not shown).

## 3. Discussion

CHDs often require complex surgeries at birth to correct the defect, and one critical predictor of survival is birth weight [2]. In pregnancies complicated by fetal CHD, abnormalities of placental development and function likely contribute to the growth restriction and prematurity that negatively impact clinical outcomes [3,4,5,10,11]. Nearly 400 genes known to be associated with CHD [17,18,19]. However, many of the more in-depth studies, aimed at determining the particular contribution of the genes or environment to cardiac defects, overlook the potential disruption to extraembryonic development that is crucial for maintaining the appropriate in utero environment and fetal organ development and growth. In the present study, we show the effect of targeted loss of *Hand1*, a gene known to be crucial in heart development, on placental development throughout gestation. Targeted loss of *Hand1* in chorionic and labyrinthine progenitor trophoblasts at E8.5-9.5 led to abnormal formation of the placental labyrinth from both a trophoblast and endothelial perspective, ultimately contributing to embryonic lethality from E12.5. Interestingly, the loss of *Hand1* in labyrinthine endothelial cells from E12.5 resulted in reduced vascular expansion and remodeling associated with reduced fetal and placental weights, and elevated risk of fetal demise by near-term.

*Hand1* has been shown to be expressed during the differentiation of human trophoblast stem cells to syncytiotrophoblast cells in early pregnancy [33]. Interestingly at term, neither isolated human placental cytotrophoblasts nor villous tissue express HAND1 [34]. However, no studies have identified if vascular remodeling throughout gestation requires HAND1. In our present study, targeted early pregnancy loss of *Hand1* in chorionic and labyrinthine progenitor trophoblasts resulted in embryonic lethality after E12.5. Co-expression of the Nkx2-5 reporter and Hand1 was limited to labyrinthine progenitor trophoblasts at gestation day 8.5 and expanded to include chorion by E9.5 and syncytium by E10.5. It was previously demonstrated that in *Hand1*-null mice, trophoblast stem cells fail to differentiate into trophoblast giant cells (TGCs) and exhibited reduced invasion, failed placentation and fetal demise around E6.5 [35], however in our study, *Hand1* expression was maintained in the TGCs of *Nkx2-5^Cre^;Hand1^A126fs/+^* placentas allowing us to study labyrinth components and development. Our histological analyses indicate that *Nkx2-5^Cre^*-driven disruption to *Hand1* does not significantly impact differentiation of the trophoblast stem cells into TGCs (sinusoidal, canal, and parietal) for establishment of the placenta, but does disrupt labyrinth formation and syncytialization. In mice, at E10-10.5 there is a switch in nutrient and oxygen supply mechanisms whereby support for continued embryonic survival changes from histiotrophic to hemotrophic [36]. In the case of the *Nkx2-5^Cre^;Hand1^A126fs/+^* placentas, failure to form the appropriate layers of the exchange area of the placenta within the labyrinth (syncytium and vasculature) by E10/10.5 ultimately prevented this switch. While subsets of trophoblast cells within the labyrinth were still capable of receiving oxygen and nutrients carried in blood into maternal blood spaces, failure of syncytial layers and inadequate vascularization of the labyrinth resulted in a failure to transfer nutrients and oxygen to the fetal circulation, resulting in fetal demise.

Similar to targeted loss of *Hand1* in early pregnancy, targeted loss in the placental endothelial cells after E12.5 resulted in disrupted placental vascular expansion and remodeling. Altering *Hand1* expression in endothelium did not appear to cause early fetal loss, as survival at E12.5 was not significantly lower than the spontaneous loss levels in this mouse strain. However, later-gestation fetal demise was significantly higher in the *Cdh5^Cre+^;Hand1^A126fs/+^* fetuses compared to wildtype littermates, pointing to a role for *Hand1* in placental vascular remodeling and expansion of the placental labyrinth. The expansion of the labyrinth and increase in branching of the labyrinthine vasculature is vital to support the exponential fetal growth phase of late pregnancy and it’s failure, as we observed from E16.5 in the *Cdh5^Cre+^;Hand1^A126fs/+^*, leads to impaired fetal growth and in some cases late term fetal demise. Despite no differences in placental fetal vessel density at E16.5, we did identify reductions in the expression of *Angpt1* and *Tie2*. *Angpt1* is a growth factor which predominantly acts on endothelial cells, and whose signaling through its receptor *Tie2* is capable of various vascular shaping functions including maintaining vascular integrity, vessel remodeling, cell migration and as anti-inflammatory potential [37]. Further studies, beyond the scope of the present one are required, but reduced expression of *Angpt1* and *Tie2*, as well as reduced labyrinthine vessel density at E18.5 does provide strong evidence that *Hand1* expression in placental endothelial cells is required for the angiogenic remodeling required to support fetal growth in late pregnancy.

Complete knockout of *Hand1* results in embryonic lethality [23,24,25,26] and given that deletion of *Hand1* in only cardiomyocytes results in fetal survival to term with only mild cardiac phenotypes [28], this suggests that the embryonic lethality is not solely due to issues with cardiac development. Results of the current study support this hypothesis; that abnormal placental development due to *Hand1* disruption plays a primary role in the fetal demise in the *Hand1*-null mouse model. The role that the placenta may play in contributing to, or exacerbating the development of CHD, remains understudied. Many genes previously associated with CHD [17,20] have not been adequately investigated in placental development or function. The Deciphering the Mechanisms of Developmental Disorders (DMDD) mouse screen [22], and our study of placental gene expression in human CHD placenta samples identifying multiple ‘heart-specific’ pathways [15,38], underscore the importance of understanding the roles of developmental genes shared between placenta and heart. The dual impact of a genetic disruption to the development of embryonic (heart) and extra-embryonic (placenta, yok sac) organs, plus in utero environmental disruptions in oxygen and nutrient supply secondary to abnormal placenta development, may provide mechanisms that underlie early fetal loss, and most importantly those that underlie the high rate of miscarriage in humans associated with CHD directly or in prior/future pregnancy [39].

This study presents clear evidence that placental development is critical in driving fetal growth and survival in the context of CHD. One limitation to the study is that mouse and human placenta structure and development differs substantially [40], hence there are implications for the translatability of the findings to human pregnancy; although the placenta cell types in which *Hand1* was manipulated are present in both mouse and human placenta. Additionally, the manipulation of *Hand1* expression occurred either before or after establishment of the placental labyrinth (E10.5) and as such, future studies are required in order to fully understand the implications at this critical time in placental development.

In conclusion, by assessing placental development in the setting of a previously developed *Hand1* mutation known to result in cardiac defects [28], we have begun to explore the mechanisms that may result in adverse pregnancy outcomes in the setting of CHD. Disruption of critical stages in placental development and function could contribute to the known clinical observations of stillbirth, fetal growth restriction, and prematurity. Our data highlights the necessity for future research to not only consider the contribution of genetic manipulation to the organ of interest but also the extraembryonic tissue and the in utero environment resulting from its disruption. By doing so, we can then better understand the broad overlap of placental and cardiac development which may ultimately drive novel therapies to improve outcomes for children with CHD.

## 4. Materials and Methods

### 4.1. Animal Procedures

All animal procedures were performed under protocols approved by the Institutional Animal Care and Use Committee of CCHMC (IACUC 2018-0065, 12 November 2018). Mice were housed and maintained under temperature and humidity-controlled environments on a 12:12 h light cycle with water and food provided ad libitum.

### 4.2. Cre Expression and Validation

To verify to efficacy and spaciotemporal expression of the *Nkx2-5^IRESCre^* (Jackson Laboratory; 024637) [31] and *Cdh5^cre^* (Jackson Laboratory, 017968), homozygous *Nkx2-5^IRESCre^* males or *Cdh5^Cre^* males were crossed with homozygous B6.Cg-*Gt(ROSA)26Sor^tm14(CAG-tdTomato)Hze^*/J female mice (tdTomato: Jackson Laboratory; 007914). Fetoplacental units were then collected at E8.5, E9.5 and E10.5 for the *Nkx2-5^Cre^*, and E14.5 and E16.5 for the *Cdh5^Cre^*, and processed for cryopreservation and frozen-sectioning. Placentas and embryos were fixed in 4% paraformaldehyde (PFA) with 2.5% polyvinylpyrrolidone (PVP), in phosphate-buffered saline (PBS) for 4 h at room temperature on a rocker plate. Cryoprotecting was achieved by rinsing the fixed tissue in PBS before placing in a 30% sucrose solution until fully infused, then embedding the tissue in OCT and storing at −80 °C. At time of sectioning, blocks were warmed to −20 °C and 7 µm sections were obtained using a cryotome (Leica, Wetzlar, Germany) before being mounted onto slides for immunofluorescent analysis.

### 4.3. Mice Mating and Genotyping

Conditionally activated *Hand1^A126FS/+^* [28] females were time mated with homozygous *Nkx2-5^IREScre^* males by placing the breeding pair together overnight and the following morning was designated GD0.5. The mating strategy produced litters containing *Nkx2-5^cre^*;*Hand1^A126FS/+^* and *Nkx2-5^cre^*;*Hand1^+/+^* embryos. For the Cdh5 study, conditional activated *Hand1^A126fs/+^* females were time mated to hemizygous *Cdh5^cre^* males to produce *Cdh5^Cre+^;Hand1^A126fs/+^*^,^ *Cdh5^Cre+^;Hand1^+/+^*, *Cdh5^Cre−^;Hand1^A126fs/+^*, and *Cdh5^Cre−^;Hand1^+/+^* fetuses. Females were sacrificed via carbon dioxide (CO_2_) asphyxiation and embryos and placentas weighed and collected at E8.5, E9.5, E10.5, E12.5, and E14.5 (Nkx2-5 study) and E12.5, E16.5 and E18.5 (Cdh5 study). At E8.5 and E9.5, implantation sites were collected, fixed and paraffin embedded for histological analysis. At E10.5 to E18.5, placentas were halved with one half fixed for histology and the other flash-frozen in liquid nitrogen and stored at −80 °C for molecular analyses.

Genotyping was performed on all feto-placental units within the litter by removing part of the yolk sac (E8.5, E9.5 and E10.5) or clipping the tail of fetuses (E12.5, E14.5, E16.5 and E18.5). Tissue was digested in 72.75 µL Direct PCR lysis buffer and 2.25 µL Proteinase K for 4 h at 56 °C, vortexed, then denatured for 30 min at 85 °C. PCR was then performed on 1 µg extracted DNA using FastStart PCR Master (Roche) and primers specific to *Hand1* (Supplementary Table S1) under the following conditions: 95 °C for 4 min, 35 cycles of 95 °C × 30 s, 65 °C × 30 s, 72 °C × 1 min, and 72 °C for 7 min. To detect the frameshift mutation at the *Hand1* locus, the *Hand1* PCR product was digested with the restriction enzyme FauI (R0651, New England Biolabs, Ipswich, MA, USA) following standard protocol using 1 µL of restriction enzyme with 1.5 µL of PCR product, 2 µL CutSmart buffer, and 20 µL RNAse-free water and visualized on a 1% agarose gel. Fetal sex determination was performed using PCR following the protocol outlined in [41] with primers provided in Supplementary Table S1.

### 4.4. RNA Expression via Reverse Transcription Quantitative Polymerase Chain Reaction(qPCR)

For RNA extraction and qPCR gene expression, flash-frozen half placentas at E10.5 (Nkx2-5 study) and E16.5 (Cdh5 study) were placed into RLT lysis buffer (Qiagen, Hilden, Germany) and homogenized. RNA was extracted using a RNAEasy Mini kit (Qiagen) following standard protocol, and quantification and quality control assessed using Nanodrop Spectrophotometer (Thermo Fisher, Waltham, MA, USA). From each sample 1 µg of RNA was converted to cDNA using the Applied Biosystems high-capacity cDNA conversion kit per protocol. cDNA was stored at −20 °C. mRNA expression levels of *Angpt1*, *Angpt2*, *Vegfα*, *Plgf* and *Tie2* were assayed in 25 µL SYBR Green (Applied Biosystems, Waltham, MA, USA) PCR Master Mix reactions containing 1/40th of the cDNA template and 300 nm/L of forward and reverse primer (Table S1). qPCR was performed in triplicate using the Applied Biosystems StepOne Plus Real-Time PCR System. Expression was normalized using the housekeeping gene *Rps20*. Relative quantification expression levels were calculated by comparative CT method using StepOne software v2.3 (Applied Biosystems).

### 4.5. Immunohistochemistry and Immunofluorescence

For paraffin-embedded implantation sites at E8.5 and E9.5, blocks were systematically sectioned until mid-sagittal placenta tissue could be identified and then 5 µm serial sections were obtained. Paraffin-embedded placenta tissue at E10.5 through to E18.5 were also serial sectioned at 5 µm. To assess gross morphology at E9.5 hematoxylin and eosin staining was performed. Briefly, sections were deparaffinized, rehydrated, placed in hematoxylin for 45 s, washed in running tap water, placed in 80% ethanol, dipped 3 times in eosin followed by dehydration, clearing and mounting in xylene-based mounting medium.

Immunohistochemistry for Hand1 was performed on sections of mouse placenta at E9.5 in the Nkx2-5 study, and for CD-31 at E10.5 and E12.5 in the NKx2-5 study and at E16.5 and E18.5 in the Cdh5 study. Sections were deparaffinized, rehydrated, and antigen retrieval was performed using Target Retrieval Solution (Dako, Santa Clara, CA, USA). Endogenous peroxidase activity was blocked using 3% hydrogen peroxide. Nonspecific binding was blocked with a 10% serum solution corresponding to the secondary antibody host species followed by overnight incubation with the primary antibody (antibodies and dilutions outlined Supplementary Table S1). Sections were then washed and incubated with appropriate biotinylated secondary antibodies. Antibody binding was amplified using the avidin-biotin complex (ABC) kit (Vector Laboratories, Inc., Burlingame, CA, USA) and visualized using the enzyme substrate DAB (Vector Laboratories, Inc.). Sections were counterstained briefly in hematoxylin before dehydration, clearing and mounting. Protein localization was observed by light microscopy (Nikon, Tokyo, Japan). In order to assess the number of fetal vessels within the placental labyrinth, two CD-31 stained sections from each placenta were analyzed. Ten high-powered images at least 50 µm apart across the placental labyrinth were obtained, the number of fetal vessels counted manually and then averaged to obtain a number per high powered field (HPF).

To verify the efficacy and spaciotemporal expression of Nkx2-5 and Cdh5, placenta samples from pregnancies between *Nkx2-5* and *Cdh5* males and tdTomato reporting females were stained for Hand1 or cytokeratin 7 (*Nkx2-5* study only) using immunofluorescence. Sections were treated as described for immunohistochemistry, incubated with an appropriate fluorochrome-conjugated secondary antibody and nuclei counter stained with Dapi. For the Nkx-2-5 study, representative images were obtained using the Nikon Eclipse 80i fluorescent microscope. For the Cdh5 study, representative images were obtained using the Axioscan (Zeiss, Oberkochen, Germany).

To assess placental microstructure in the Nkx2-5 study, double-label immunofluorescence for cytokeratin 7 and CD-31 was performed on sections of mouse placental at E9.5, E10.5 and E12.5. Sections were treated as described for immunohistochemistry until the primary antibody was applied. Following, primary antibody incubation, sections were washed, incubated with appropriate fluorochrome-conjugated secondary antibodies and counterstained with DAPI before being mounted with antifade mounting media. Fluorescence microscopy was performed using the Nikon Eclipse 80i fluorescent microscope.

### 4.6. Statistical Analysis and Data Presentation

Statistical analysis was performed using Prism GraphPad (v8.4.3). Shapiro–Wilk test was used to test for normality in the data. Statistical significance in data which passed the normality test was determined using student’s *t*-test and expressed as mean ± SEM. Statistical significance in data which did not pass the normality test was determined using Mann–Whitney test and expressed as median ± interquartile range. n number refers to the number of litters. Level of significance (*P*-value) was defined as *P* ≤ 0.05.

## Data Availability

Data supporting these results can be provided upon request to corresponding author.

## Supplementary Materials

The following are available online at https://www.mdpi.com/article/10.3390/ijms22179532/s1.
