# Supplementary material for: Conditional Mutation of Hand1 in the Mouse Placenta Disrupts Placental Vascular Development Resulting in Fetal Loss in Both Early and Late Pregnancy
Source: Int J Mol Sci. 2021 Sep 2;22(17):9532. doi: 10.3390/ijms22179532 (PMC8431056; doi:10.3390/ijms22179532)
Supplement: Supplementary file 1 [file ijms-22-09532-s001.zip › ijms-1309477-supplementary.pdf]

**Supplementary Material**

**Table S1** Genotyping primer sequences, qPCR primer sequences and immunohistochemistry/immunofluorescent antibodies

| **Genotyping Primers** | **Forward** | **Reverse** |
|---|---|---|
| Hand1 275-448 | 5’-GCCCAAACGAAAAGGCTCAG-3’ | 5’-AGCACGTCCATCAAGTAGGC-3’ |
| Sry | 5’-AACAACTGGGCTTTGCACATTG-3’ | 5’-GTTTATCAGGGTTTCTCTCTAGC-3’ |
| Nifα | 5’-TGCTGTGTTCTGGTCAGTCAAG-3’ | 5’-CAAAGCAAATCTCCATGCTCGG-3’ |
| | | |
| **qPCR Primers** | **Forward** | **Reverse** |
| Angiopoietin 1 | 5’-GCACGAAGGATGCTGATAAC-3’ | 5’-AACCACCAACCTCCTGTTAG-3’ |
| Angiopoietin 2 | 5’- GCACAAAGGATTCGGACAAT-3’ | 5’- AAGGACCACATGCGTCAAA-3’ |
| Vascular endothelial growth factor α | 5’-TTAAACGAACGTACTTGCAGATG-3’ | 5’- AGAGGTCTGGTTCCCGAA-3’ |
| Placenta growth factor | 5’- GACCTATTCTGGAGACGACA-3’ | 5’- GGTTCCTCAGTCTGTGAGTT-3’ |
| Angiopoietin 1 Receptor (Tie2) | 5’-GATTTTGGATTGTCACGAGGTCAA-3’ | 5’-CACCAATATCTGGGCAAATGATGG-3’ |
| | | |
| **Antibodies for IHC and IF** | **Source** | **Concentration** |
| Hand1 goat | R&D Systems AF3168 | 1:100 |
| CD-31 (IF) goat | R&D Systems AF3628 | 1:100 |
| CD-31 (IHC) rabbit | Abcam ab28364 | 1:100 |
| Cytokeratin 7 rabbit | Abcam ab199718 | 1:800 |
| Biotinylated anti-Goat | Vector BA-5000 | 1:200 |
| Biotinylated anti-Rabbit | Vector BA-1000 | 1:200 |
| Alexa Fluor 488 anti-Goat | Invitrogen A11078 | 1:200 |
| Alexa Fluor 647 anti-Rabbit | Invitrogen A32795 | 1:200 |
| Alexa Fluor 555 anti-Goat | Invitrogen A27017 | 1:200 |
